# Technic for the Removal of Dead Teeth

**Published:** 1919

**Authors:** 


**Technic for the Removal of Dead Teeth.** By J. Novitzky. *The American Journal of Surgery,* March 1918.

The author speaks of the dangerous septic conditions in the jaws due to dead or devitalized teeth, and insists upon the fact that dead teeth should be removed. Dead teeth and disintegrated bone at their root ends cannot be effectively treated and drained through the root canals of the teeth. Putrefactive changes may be retarded and both the tooth and the area filled with granulations and different strains of micrococci rendered temporarily passive but never really cured. Dead teeth like other dead organic structures are subject to the laws of decomposition. They may serve as organs of mastication, but they should not be retained in the jaws because they are a menace to health and even to life. Dead teeth should not be removed by the old method of extraction. Extraction generally fails to eradicate the diseased area; it commonly causes fractures of the alveolar process or of the tooth itself; it may fail to remove broken down alveolar process or granulations; it may fail to disclose complications and also to remove all the pathological area. Infected dead teeth and alveolar process should be removed by a surgical dissection, i. e. as follows:

In order to gain space it may be necessary to strip down the gums and periosteum from the healthy tooth on each side of the necrotic one. The gum is sutured back at the end of the operation. It may be only necessary to raise and pull back a triangular flap with its apex at the gingival margin of the necrotic tooth so as to expose the outer plate of bone for over the length of the tooth. When this is done, part of the outer plate is removed with a chisel so as to expose the cancellous bone and the root. If the part of the buccal plate which is removed is not so long as the root of the tooth, the latter is hooked out sidewise before the apical examination is made and the curettement done. In the contrary case, the tooth is hooked sidewise either before or after the apical exploration and curettement. The region is then blocked off by sterile sponges. When a cavity is discovered beneath the tooth roots, the tooth and the alveolar septum are removed with a chisel. The inner plate of the jaw is left intact. When the antrum is affected, the inner plate may sometimes be cut away. Antrum incisions are sutured, and the antrum must not be packed with gauze unless hemorrhage is excessive. 5 or 6 days after the operation the cavity may be irrigated. The operation may be performed under novocain anesthesia without pain and but little shock.

Five distinctive advantages of the operation here advocated are:

(1) It never results in fractures.

(2) The operator has direct access to the diseased bone.

(3) The operation discloses any pathological conditions of the nasal floor, any vents under the antral membrane, any vents into the nasopalatine canals, and into the dental canal of the mandible.

(4) The operation prevents the extensive absorption of the alveolar process.

(5) It allows the filling of the bone cavity with the gum and periosteum flap, which favors rapid healing.

